# Evaluation of Two Mobile Health Apps in the Context of Smoking Cessation: Qualitative Study of Cognitive Behavioral Therapy (CBT) Versus Non-CBT-Based Digital Solutions

**DOI:** 10.2196/mhealth.9405

**Published:** 2018-04-18

**Authors:** Carina Tudor-Sfetea, Riham Rabee, Muhammad Najim, Nima Amin, Mehak Chadha, Minal Jain, Kishan Karia, Varun Kothari, Tejus Patel, Melanie Suseeharan, Maroof Ahmed, Yusuf Sherwani, Sarim Siddiqui, Yuting Lin, Andreas B Eisingerich

**Affiliations:** ^1^ Digital Therapeutics London United Kingdom; ^2^ Barts and The London School of Medicine and Dentistry London United Kingdom; ^3^ Department of Medicine Faculty of Medicine Imperial College London London United Kingdom; ^4^ Imperial College Business School Imperial College London London United Kingdom

**Keywords:** smoking cessation, mHealth, mobile health, health behavior change, cognitive behavioral therapy, public health, health policy

## Abstract

**Background:**

Mobile health (mHealth) apps can offer users numerous benefits, representing a feasible and acceptable means of administering health interventions such as cognitive behavioral therapy (CBT). CBT is commonly used in the treatment of mental health conditions, where it has a strong evidence base, suggesting that it represents an effective method to elicit health behavior change. More importantly, CBT has proved to be effective in smoking cessation, in the context of smoking-related costs to the National Health Service (NHS) having been estimated to be as high as £2.6bn in 2015. Although the evidence base for computerized CBT in mental health is strong, there is limited literature on its use in smoking cessation. This, combined with the cost-effectiveness of mHealth interventions, advocates a need for research into the effectiveness of CBT-based smoking cessation apps.

**Objective:**

The objective of this study was, first, to explore participants’ perceptions of 2 mHealth apps, a CBT-based app, Quit Genius, and a non-CBT-based app, NHS Smokefree, over a variety of themes. Second, the study aimed to investigate the perceptions and health behavior of users of each app with respect to smoking cessation.

**Methods:**

A qualitative short-term longitudinal study was conducted, using a sample of 29 smokers allocated to one of the 2 apps, Quit Genius or Smokefree. Each user underwent 2 one-to-one semistructured interviews, 1 week apart. Thematic analysis was carried out, and important themes were identified. Descriptive statistics regarding participants’ perceptions and health behavior in relation to smoking cessation are also provided.

**Results:**

The thematic analysis resulted in five higher themes and several subthemes. Participants were generally more positive about Quit Genius’s features, as well as about its design and information engagement and quality. Quit Genius users reported increased motivation to quit smoking, as well as greater willingness to continue using their allocated app after 1 week. Moreover, these participants demonstrated preliminary changes in their smoking behavior, although this was in the context of our limited sample, not yet allowing for the finding to be generalizable.

**Conclusions:**

Our findings underscore the use of CBT in the context of mHealth apps as a feasible and potentially effective smoking cessation tool. mHealth apps must be well developed, preferably with an underlying behavioral change mechanism, to promote positive health behavior change. Digital CBT has the potential to become a powerful tool in overcoming current health care challenges. The present results should be replicated in a wider sample using the apps for a longer period so as to allow for generalizability. Further research is also needed to focus on the effect of greater personalization on behavioral change and on understanding the psychological barriers to the adoption of new mHealth solutions.

## Introduction

### Background

There is general consensus worldwide that health care organizations have historically struggled to embrace the use of information technology to increase productivity and the quality of care delivered [1]. However, the technological landscape is rapidly being transformed with the emergence of the digital revolution. A number of leading health care organizations worldwide have begun to exploit some of the opportunities for novel health care solutions [2,3]; however, there appear to be many further opportunities to be unlocked in this space, in particular in the context of digital mobile technologies.

Smartphones are considered to be one of the 8 key technologies contributing to the digital revolution [4]. The number of smartphone users has been increasing rapidly. In 2014, there were 1.57 billion smartphone users worldwide, and this is predicted to increase to 2.87 billion by 2020; the upshot is that in 2017, 96% of UK respondents aged between 16 and 34 years reported owning a smartphone [5].

Mobile health (mHealth) is a critical part of the digital transformation of health care. The Global Observatory for eHealth defined mHealth as medical and public health practice supported by mobile devices such as mobile phones, patient monitoring devices, personal digital assistants, and other wireless devices [6]. Technological advances, coupled with the unique ability of mobile apps to reach all smartphone owners at a relatively low cost, have accelerated the market growth for mHealth apps [7], such that in 2016, there were more than 259,000 mHealth apps available on the major app stores. It is predicted that by 2020, 2.6 billion people will have downloaded an mHealth app at least once—551 million of these will be active users [8].

mHealth apps can offer a number of benefits for users, such as improved treatment accessibility, real-time symptom and activity monitoring, treatment progress tracking, personalized feedback, motivational support, portability, and flexibility [9,10]. They seem to represent a feasible and acceptable means of administering health interventions [11] and have the potential to be effective in eliciting health improvements in conditions ranging from diabetes [12] to depressive symptoms [13]. Conversely, there are also a number of notable drawbacks of mHealth apps that need to be considered. These include technical problems, data security, patient privacy, timely management of assistance from a medical professional [14], as well as psychological barriers to adoption and effective user engagement.

The World Health Organization reported in 2017 that noncommunicable diseases (NCDs) are the cause of 70% of all global deaths. Cardiovascular disease, cancer, respiratory diseases, and diabetes are the biggest contributors to NCD deaths equating to 81% thereof. Risk factors such as frequent tobacco use, alcohol abuse, poor diet, and physical inactivity increase likelihood of NCDs [15]. A number of smartphone apps address these issues using behavioral change mechanisms to modify behavior and promote a healthier lifestyle.

The cognitive behavioral therapy (CBT) model is based on the idea that thoughts, emotions, and behavior interact with and influence each other. CBT is commonly used in the treatment of mental health disorders especially because of its ability to alleviate distress caused by unhelpful cognitions and reframe these cognitions to lead to more adaptive behaviors [16]. This type of psychotherapy has been incorporated within clinical guidelines because of its strong evidence base [17], which supports the idea that it represents an effective method to elicit health behavior change [18-20]. Importantly, with nearly three-fourths of current smokers reporting that they wanted to give up smoking [21], CBT has also shown to be effective in smoking cessation [22,23].

The evidence base for the efficacy of computerized low-intensity psychological CBT interventions for anxiety and depression is particularly strong [24,25]. Moreover, evidence has shown that, alongside CBT provided through a computer-based platform, CBT delivered via mobile apps could significantly improve outcomes for patients [26]. However, limited research on its use in smoking cessation exists. This, combined with the cost-effectiveness of this intervention, advocates a need for research investigating its effectiveness in this context.

### Objectives

The purpose of this study was, therefore, first, to explore users’ perceptions of two mHealth apps, one CBT-based app, Quit Genius (QG), and one non-CBT-based app, National Health Service (NHS) Smokefree (SF), over a variety of critical themes. Second, the study also sought to investigate the perceptions and health behavior with respect to smoking cessation for users of each app. To do so, a qualitative short-term longitudinal study was conducted based on semistructured interviews with users, followed by a thematic analysis, which resulted in several higher themes and subthemes. Descriptive statistics regarding participants’ willingness to continue using each of the apps, as well as perceptions and health behavior in relation to smoking cessation, were also calculated.

## Methods

### Participants

A total of 45 participants were recruited for the study, to account for any dropouts. This sample size was chosen in accordance with the recommendations for qualitative studies present in the literature, which indicate a sample size of 5 to 50 [27] to achieve saturation of results [28]. The following inclusion criteria were used: (1) smoker who intends to quit, (2) Apple iPhone smartphone user, (3) access to English App Store, (4) English speaker, (5) age >18 years, (6) has mental capacity, (7) has some experience/knowledge regarding mobile apps. Application of the inclusion criteria led to an initial sample of 45 users. Users were randomly allocated to one of 2 apps, resulting in 18 users allocated to QG and 27 users allocated to SF.

Three participants dropped out of the QG arm, and 13 dropped out of the SF arm. The final sample was thus composed of 15 participants in the QG arm and 14 in the SF arm. Demographic data of the final sample of participants were also collected (Table 1).

The NHS/HSC Research and Development Offices granted ethics approval. Fully informed written consent was gained from all participants before interview.

### Study Design

A qualitative short-term longitudinal study based on one-to-one semistructured interviews with users allocated to one of the 2 selected apps was conducted. The literature investigating the use of CBT in smoking cessation, specifically via a mobile digital platform, is limited. Due to this, an exploratory approach was used, setting out to gather data around the topic, the analysis of which would facilitate the emergence of research questions and theory. Thus, a qualitative, inductive approach was adopted. From the primary data collected, thematic analysis was carried out using a 6-phase framework [29]. This was used to generate themes from ideas that emerged during the interviews. Descriptive statistics based on quantitative data gathered as part of the interviews are also provided.

### Apps

**Figure 1:**
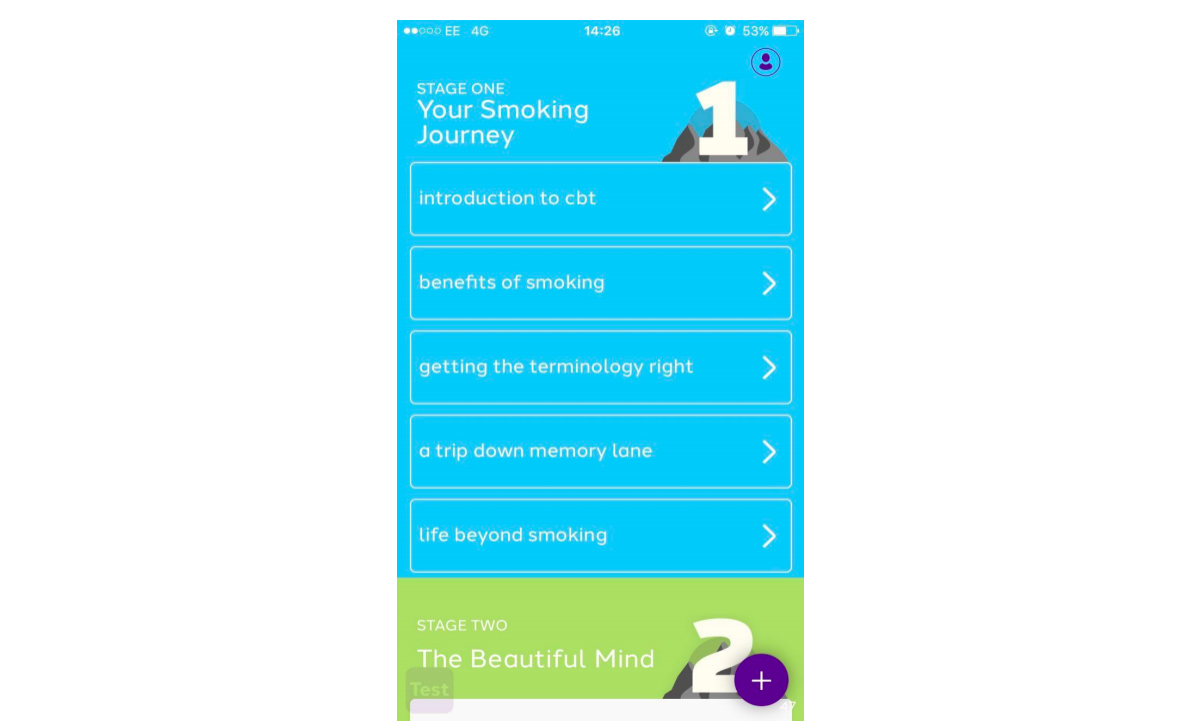
Screenshot of the Quit Genius interface. CBT: cognitive behavioral therapy.

**Figure 2:**
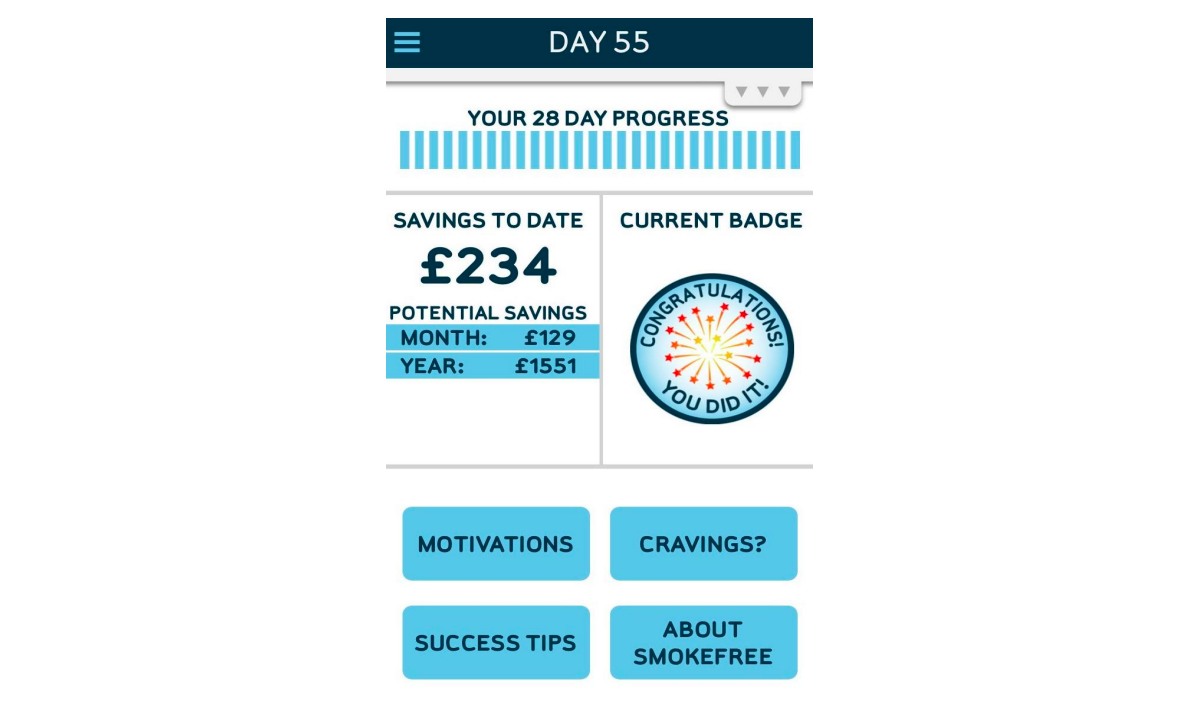
Screenshot of the NHS SmokeFree interface.

The rationale behind the choice of apps was to compare a smoking cessation app that uses CBT against one that does not. Therefore, QG (Figure 1) and NHS SF (Figure 2) were chosen. Figures 1 and 2 represent screenshots of the app interfaces at the time of the data collection. Both apps are gamified, smoking cessation, smartphone apps and offer a number of different features (see Table 2). Gamification refers to the introduction of game-like elements and principles to nongame contexts, with a view to encouraging participation and involvement [30,31].

### Interview Procedure

Each participant was interviewed twice, 1 week apart, before and after they had used their allocated app. Interviews included mostly qualitative, but also a small number of quantitative (ie, 1-10 rating scales) elements. The first interview provided a short, baseline assessment of the individual’s smoking habits and history, as well as of their perceptions of digital therapeutics and mobile apps in health care. Participants were also given standardized instructions regarding app use. Interviewers were instructed to neither encourage nor discourage the participants’ smoking behavior, to minimize bias. 

**Table 1:** Demographic data of the final sample of participants. NHS: National Health Service.

Characteristics		Quit Genius users	NHS Smokefree users
Number of participants, N		15	14
Mean age, in years		25.07	24.21
**Gender, n**			
	Male	13	12
	Female	2	2
**Occupation, n**			
	Student	11	8
	Employed^a^	4	6
**Cultural background, n**			
	Caucasian	3	9
	Asian	12	4
	British Arab	0	1
Quitting for the first time, n		6	6
Average number of cigarettes per day		7.9	7.8
Average number of times participant opened app between interviews 1 and 2		9.2	6.1

^a^PhD students were included in the “Employed” category as their financial status and daily working schedule is closer to being employed than being an undergraduate student.

**Table 2:** Characteristics and features offered by the Quit Genius and Smokefree apps. CBT: cognitive behavioral therapy; NHS: National Health Service.

Characteristics/features	Quit Genius	NHS Smokefree
Length of program	8 weeks	4 weeks
Characteristics of program	CBT-based, customized, personalized; involves the following: self-reflection, changing unhelpful thinking patterns and behaviors, development of personal coping strategies, problem-solving, and mindfulness	Non-CBT-based, involves the following: support messages to increase motivation, practical support, encouragement, tailored advice, and success tips and content
Features	Four-stage “journey” with several steps per stage, involving audio sessions and transcripts, quizzes, and interactive exercises, as well as a smoking diary	Daily support messages; badges rewarding progress, craving button with tips and content, savings calculator, personalized motivation; success tips
Gamification elements	Presenting the program as a journey with achievements, progress bars, time-based challenges	Progress tracking, badges rewarding progress, and savings calculator

The second interview assessed users’ evaluation of their allocated app, as well as the effects the use of each app had on smoking-related perceptions and behaviors.

Interview questions were initially prepared, then piloted using 4 independent participants. Adjustments to the interview questions were made based on findings from these pilot interviews. The pilot study confirmed the suitability of the 2 chosen apps. The results of the pilot were not included in the final study.

Verbal consent was gained from all participants to record interviews on a smartphone or laptop software.

### Thematic Analysis

Braun and Clarke’s [29] 6-phase framework was used for our thematic analysis. An inductive approach was adopted during the coding process; all concepts and ideas that arose were coded, regardless of relevance to the original research question. A manual coding process was undertaken. The transcripts were printed, and individual codes were highlighted and transferred onto post-it notes, in the process of identifying segments of the data. The post-it notes were color-coded to facilitate easy visualization of codes for the development of themes. Once coded, each transcript was then reread by a second researcher to check the rigor of coding and to increase the conformability of the codes created. The codes were analyzed to form overarching themes, and ambiguities were resolved via discussion among members of the research team.

### Descriptive Statistics

Descriptive statistics regarding the willingness to generally use a smoking cessation mHealth app, as well as motivation to quit smoking and different aspects of smoking behavior of users of each app, were also calculated.

## Results

### Thematic Analysis

**Figure 3:**
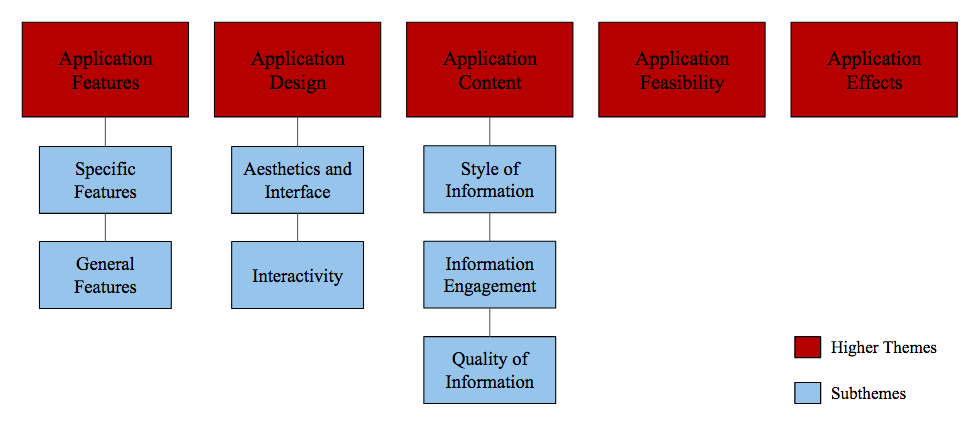
Visual representation of the five overarching themes and their respective subthemes, which resulted from the thematic analysis.

The thematic analysis resulted in five overarching (higher) themes that influenced the impact of the interventions on health behavior. Three of the higher themes were associated with several subthemes (Figure 3). The themes were explored with a view to determining the relationship and differences between the 2 apps. The five higher themes were not exclusively related to smoking cessation.

### App Features

#### Specific Features

One user commented positively on QG’s “memory lane” as their favorite feature. Multiple participants also spoke positively of the smoking diary feature that allowed them to log every cigarette they had and reminded them to open the app:

You can see the pattern of why you've been having cigarettes.

SF users spoke poorly of the “lapse” feature, as it made them feel guilty disengaging them from the app. One of the users commented:

The [SF] app makes you feel like you've failed if you relapse.

SF users were also wary of the sharing capabilities of the app, specifically with social media. They did not feel comfortable with the option, and the thought of doing so made them feel insecure. One user said:

Don't want to share progress on social media in case you fail.

Multiple users also mentioned that the SF “savings” feature was inaccurate, and this led to a lack of trust in the app, making users question the integrity of all aspects of its design. Conversely, other users appreciated the existence of the “savings” feature and used it as motivation to continue with the program.

#### General Features

Participants commented on the idea of personalization in both QG and SF. It was agreed that the ability to tailor the app to your personal situation was pivotal for engagement. One participant stated:

...it's like a personal kind of message to yourself to say...so it’s like in a motivation rather than someone else telling you what to do.

QG participants found that the QG notifications were motivational and engaging:

I liked how it gave notifications, like every day I've got a notification saying; You're on day four of your smoking quitting history. You could do this, don’t give up. Stay loyal and stuff like that. That was quite impressive.

On the other hand, a significant number of SF users commented that the SF tips functions and notifications were generic and not useful, some even choosing to ignore them all together. Some, however, found the tips function useful, helping them commit to their goals.

Users also mentioned that they enjoyed the multimedia functionality of QG. They commented particularly on the audio feature being soothing and engaging. Multiple QG users also commented on the quizzes, suggesting that they helped reinforce the knowledge that the app provided. One of the users said:

The quizzes are pretty useful and there’s a sense of achievement from getting them right.

### App Design

#### Aesthetics and Interface

The majority of participants found the QG app’s aesthetics and layout favorable, with a positive, bright color scheme. Multiple users also appreciated that the QG app did not appear too clinical:

[QG has a] simple design...it looks like a professional app.

SF was identified as aesthetically appealing by a few participants; a positive response to the color scheme was exhibited. However, other participants were less enticed by the aesthetics of SF. One of the participants commented:

[SF] made me feel childish...it’s a little bit Mickey Mouse.

A number of QG users were frustrated by a bug in the app regarding the progress bar:

[The QG] progress bar at bottom doesn't always work, [you] have to refresh the app.

Nearly, all SF participants noted that the app was very easy to use and navigate. The lack of technical issues such as bugs or slow response times resulted in minimal effects on overall experience. One user stated:

[SF is] simple, easy to use, user-friendly.

Although the QG app’s steps were quite extensive, participants felt they were well instructed and given sufficient guidance when operating the app.

On the other hand, SF participants reported that the interface provided poor instructions and guidance:

It doesn't really instruct you what to do next.

#### Interactivity

QG was found to be highly interactive, with generally positive responses regarding engagement. Participants were especially engaged by the audio narrations:

[I] felt interactive with the speaker.

Notably, this was not always observed, as a small minority of users struggled to engage with QG, expressing a sense of boredom because of perceived lack of captivating material. One of the users stated:

[I] would prefer some kind of like visual effects.

A number of participants highlighted the lack of interactivity in SF, which caused boredom and a decreased desire to use the app. One participant said:

It just needs to be made more interactive.

### App Content

#### Style of Information

Multiple participants appreciated how QG could be listened to, with audio being considered more insightful than just reading text on a screen. Some even associated the benefits of audio with convenience, as it can be carried out while performing other activities. However, some users preferred using the transcript alone as it allowed them to set their own pace. This is evident from the following statements:

I just listened to this thing...because I don’t like reading.

It’s good because it’s audio so you can just listen to it while you’re in the train...you don’t actually have to be physically on your phone.

The terminology of the information was also shown to make a difference in the effectiveness of the content. One of the participants commented:

I think a lot of the initial sessions were set out really well, so actually understanding a bit about CBT and the things about getting the terminology right, I think that’s important to understand the rest of the app.

Furthermore, there was also particular appreciation for when information was delivered concisely and not presented in large chunks, minimizing information overload:

[QG] starts and stops so you can only do progress at certain stages. That’s good, because it limits the amount of information you get all at one go.

Users of SF did not comment on the style of information, as it provided a more traditional app style.

#### Information Engagement

The majority of QG participants found the information and the introductory video, in particular, engaging:

I find it really engaging, I suppose that’s why I stuck with it.

Building on that, participants noted that when engaged, they enjoyed learning, and it helped them understand the consequences of smoking:

I...enjoy learning something new. It’s quite informative and makes you think about what you’re doing.

[QG] helps you to understand a bit more about what’s going on...what could go wrong by continuing (to smoke).

Some QG users reported difficulty in engagement as the app required time commitment for the audio features. Furthermore, repetition was conducive to loss of interest. One participant said:

Three to four minutes...doesn’t seem long at all but when you’re listening to the guy it seems on for ages.

Overall, a minority of SF participants found the SF information engaging. The participants noted that if they were engaged, they wanted to know what happened next in the app:

I was just interested to see what badge came next...that's quite interesting in itself.

However, SF users felt bored using the app:

Bit bored by it and [did] not want to use it.

#### Quality of Information

Overall, the majority of QG participants were impressed by the quality of information. The QG content went beyond what the users expected, helping them understand the consequences of smoking. It was also well received when the content was found to be relatable, as users felt a more personal connection:

It obviously isn’t a tailored app to each person, but it gives enough information that each person can relate to it in a tailored way.

Furthermore, it was also noted that when information debunked common myths, it had a larger impact on the user:

They’re talking about debunk a lot of the myths that tobacco companies put across, or corporate greed.

Several SF participants stated displeasure in the quality of information provided in the app. SF users lost interest in the app when provided with information they already knew. Poor information quality seemed to leave a lasting impression on the participants:

I think everyone has heard that information many times.

It’s actually quite patronizing...shallow stuff, not hard hitting useful facts.

### App Feasibility

Multiple users spoke favorably of the potential of mobile apps in providing therapy, commenting that apps are easy to access and use without the need for previous training or advice. The time commitment required is generally less than in the case of other treatment forms. It was further reported that mobile apps have the advantage of being low cost with a wide reach. Users also identified benefits because of the fact that one can receive treatment on one’s own terms, independently, and with an element of privacy. This is echoed by the following statements:

It’s in your pocket, easily accessible. Available to a lot of people, all the time.

Less time commitment with a greater focus on the modern use of technology. It’s the way to quit smoking that best fits into a modern person’s life.

Some users, however, identified that apps on phones are easily forgettable, and engagement often reduces over time. Moreover, the lack of human contact from a physician deemed mobile apps to be a less attractive option. One user said:

I probably wouldn’t want to use an app but would want to have personal contact with the doctor or trained physician.

### App Effects

QG users noted a significant number of *changes in their perceptions*. Most participants reported that they had changed the way they thought about smoking to some degree. Many users appreciated the purpose of CBT and valued the way that CBT provided information and tools to make their own decisions and trained the brain to think in new ways. Participants reported that the app helped them explore their own smoking journeys and was valuable in understanding psychological triggers and cues of why they smoked and reevaluate their smoking behavior:

It links your thoughts to your behaviour. It worked. It questions why you do things rather than just when you just do it you just do it.

It’s training your brain to think in new ways, to not associate certain things with certain things.

...this [QG] changes your mind.

QG increased confidence in users who had previously perceived quitting as an impossible task such that they reported that quitting now seemed more feasible. Many QG users identified how the app had improved their willpower to quit smoking. In contrast, relatively few users reported decreased motivation to quit after the week, with one user reporting they felt a lot of effort needed to be inputted to feel engaged by the app:

It has made me realise that it’s more feasible to quit. It's not impossible.

Some QG participants noted that labeling themselves as “non-smokers” as opposed to “ex-smokers” increased their confidence in their ability to quit smoking. One user stated:

Yes it has given me confidence to stop smoking and focus on being a non-smoker not an ex-smoker.

A small number of participants also displayed successful internalization as they reported that they applied a visualization exercise provided by the app in their day-to-day life. These participants emphasized that doing the exercise did not require direct access to the app. Referring to this exercise, one of the participants said:

There is an exercise that you imagine yourself as a non-smoker and that you're going to an event - and everybody there is a non-smoker and you put yourself there. I really like that bit so I can actually take that exercise in my head and do that anywhere.

The majority of participants expressed their desire to continue to use the QG app. Users reported talking about the app to friends and family:

I found myself talking about it with the people as well trying to explain to them how it describes smoking and why would you do it and what it actually chemically does you know. This is almost like you're reeling off facts but they're quite interesting.

A small number noted they were less likely to continue using the app, as mobile apps, in general, are easy to forget:

It’s not a thing that I'll remember daily.

Users also reported enjoyment in using the QG app and in learning:

I actually really enjoyed it.

A number of SF users reported that they experienced no positive behavior changes after the use of the app. Most SF users stated that they would not continue to use the app further:

I wouldn’t say it’s hugely changed my smoking habits.

One user quit smoking on the first day of the study, reporting that they felt highly motivated to stop so that they could log being “smoke free” on the app. A few participants reported that the SF app itself increased the urge to smoke, resulting in an increase in smoking:

[SF] can cause the urge to smoke.

Most SF users reported the app to be ineffective with lack of impact. The lapse feature recurrently reminding users of slips in their smoking cessation journeys resulted in negative emotions. One participant stated:

You feel a little bit like a loser.

### App Improvements

Users of both apps recommended adding more personalized features to the apps. Such features included customized motivation scales or tailoring tips and a progress monitoring feature. One user stated:

The tips are not necessarily very tailored to the person.

QG users suggested improvements to the audio clips, some requesting shorter, more concise clips, and others suggesting videos for any text-heavy topics.

Many QG users recommended the addition of an in-app forum whereby users could have the opportunity to interact with other users for motivational reinforcement. One of the participants said:

So having some sort of platform where everyone can just say, “This is how I stopped” or “This is how I'm trying to stop” and then other people giving feedback saying, “This is good” or, “This is not.”

A few participants reported that a gaming aspect in the SF app would be a desirable attribute:

Maybe if they had prior to like some type of like a mini game or something in there that would keep the mind occupied rather than telling you, “Don't smoke.”

The SF users specified several individual improvements. Visualization, such as a graphical representations monitoring health, was deemed to be a key feature of an ideal app with a number of SF users. Some users also suggested regular health news updates such as smoking taxes and bans.

### Descriptive Statistics

Users of QG were, on average, more willing to use a smoking cessation app to manage their health, in comparison to SF users (Table 3).

In addition, participants having used QG for 1 week reported, on average, several positive behavior changes, such as increased motivation to quit smoking and reduction in the number of cigarettes smoked per day (Table 4). QG participants were similarly more likely to recommend the app, compared with SF participants.

**Table 3:** Numbers of users whose willingness to use a smoking cessation app to manage their health was high, moderate, or low, for each app. NHS: National Health Service.

Willingness to use smoking cessation app to manage health	Quit Genius (N)	NHS Smokefree (N)
High (increased willingness)	10	5
Moderate (no change in willingness)	4	4
Low (decreased willingness)	1	5

**Table 4:** Overall patterns of users’ perceptions and health behavior change in relation to smoking cessation for each app.

Number of participants who:	Quit Genius, n (%)	NHS Smokefree, n (%)
Decreased number of cigarettes/day	8 (53)	2 (14)
Increased number of cigarettes/day	0 (0)	3 (21)
Showed increased motivation to quit smoking	8 (53)	5 (36)
Expressed desire to continue using app	10 (67)	5 (36)
Recommend the app	11 (73)	5 (36)

## Discussion

### Principal Findings

Five higher themes and several subthemes resulted from the thematic analysis. QG users were generally more positive and receptive with regard to the app’s features, design, as well as information engagement and quality, compared with SF users. QG users also reported changing their perceptions and way of thinking with respect to smoking. It is possible that the root of this effect may lie in CBT, which gives users the opportunity to explore and change their thoughts and perceptions related to smoking.

On average, QG users also noted an increased willingness to use a smoking cessation app in general to manage health. They also showed increased motivation to quit smoking, as well as more willingness to continue using their allocated app after 1 week. These participants also showed changes in their smoking behavior although this was in the context of our limited sample, not allowing for the finding to be generalizable as of yet.

Several findings emerged in terms of the features of the apps and their relationship to behavior change.

A change in the manner of thinking about smoking was deemed important by participants with regard to a possible change in behavior. This was prominent in QG users, and it is possible that this is because of the app’s use of CBT. Users reported that the app allowed them to question why they smoke, what smoking means to them, as well as their thoughts about quitting and why this is something they want to achieve. QG also allowed users to reframe the way they thought about themselves and their behavior in relation to smoking. For example, several QG users reported that perception-altering exercises such as labeling themselves as “nonsmokers” as opposed to “ex-smokers” helped them dissociate themselves from the behavior and contributed to a reduction in smoking in these users. No such effects were reported by the SF users.

QG users also reported that the CBT method contributed to their intrinsic motivation to quit, making them perceive themselves at the source of their decisions and therefore feel empowered to take control of their own actions in relation to their journey to smoking cessation. This coincided not only with an increase in self-efficacy, that is, one's belief in one's ability to succeed in specific situations or accomplish a task [32] but also with an increase in behavioral control, that is, the level of difficulty an individual associates with a behavior [33]. Specifically, QG users reported that they believed the app had equipped them with increased confidence in their ability to quit, making the concept of quitting seems easier, more realistic, and thus more achievable. SF users did not report such a change in their belief related to their ability to quit smoking; many noted that the advice and tips provided by the app were already known and too generic. However, some SF users noted that just by downloading the app, they felt more equipped to quit than previously.

Although users of both apps understood and reported some of the benefits of smoking cessation, such as better health and saving money, SF users mostly felt that their knowledge was left unchanged, as the information provided, for example, regarding the harms of smoking, was generic and well known. Therefore, this had no impact on their understanding of the consequences of smoking. Conversely, QG users were positive about the effect on their knowledge, mentioning that reinforcement of the consequences at multiple points during the progress gave them greater motivation to quit smoking. This fits well with the health belief model [34-36], in which the perceived threat (in this case, the health hazards associated with smoking) plays a vital part in the individual’s likelihood to engage in health-promoting behavior. Generally, users reported feeling bored using SF, which provided information already known to the users, whereas QG was seen as novel and informative. This is not surprising, as implementing CBT in such a gamified app is a new concept.

Users also highly commended QG for not using scare tactics to drive change in behavior but instead supporting and guiding users gently through the process. This goes against common literature that suggested fear-appeal and antismoking tactics are effective in promoting smoking cessation [37-39]. A possible explanation in this study may be related to the fact that a large proportion of our participants were relatively young and, therefore, identified scare tactics as an out-of-date strategy, preferring to be given the information and opportunities to make their own decisions. Another reason may be that scare tactics make reference to information that is already vastly known to people about the dangers of smoking, but these tactics do not acknowledge the great difficulties associated with nicotine addiction and fail to provide practical support.

### Implications to Practice and Barriers to Implementation

The findings of this study suggest that a mobile app based on CBT was favorably perceived by users in terms of features, design, as well as information engagement and quality, in the context of smoking cessation. This was associated with changes in users’ perception and thinking manner with regard to smoking. On average, users of the gamified CBT-based app also showed increased willingness to use a smoking cessation app, in general, to manage health, as well as increased motivation to quit smoking and positive changes in smoking behavior. A non-CBT-based mobile app was less favorably perceived, yet some users viewed some features and the app’s interface as useful. Other apps based on therapeutic principles such as acceptance and commitment therapy (ACT), which has common elements with CBT, have also been developed and shown to be effective in smoking cessation [40].

Given the significant estimated smoking-related cost to the NHS (£2.6bn in 2015; [41]), the possibility of using mobile apps to influence health behavior may have implications in the current economic climate of health care, contributing to the growing use of mobile apps in this domain. Specifically, exploiting such advances in technology could contribute to the needed efficiency savings of 2% to 3%, compared with the current 0.8%, noted in the NHS England Five Year Forward Review, in the context of the £30 billion funding gap predicted to occur by 2020/2021 [42].

Further advantages of apps include opportunities for scalability across the NHS and eliminating postcode lottery issues, as well the possibility of increased adherence to interventions because of the convenience of use. Indeed, the review suggested expanding the set of NHS-accredited health apps available to patients [43], whereby apps may even be prescribed as treatment or part thereof in the future.

However, reengineering processes to implement mHealth apps into daily medical practice, such as first-line treatment recommendations, will involve a rigorous change management process. A crucial aspect of this is ensuring that apps are compliant with privacy standards, as illustrated by the release and subsequent withdrawal of the NHS mHealth Applications Library pilot in 2013 because of noncompliance. mHealth apps have the capability to collect a vast amount of data, which can then be used to improve medical care in the future via predictive analytics and artificial intelligence. However, extensive security and privacy systems need to be put in place before apps can be confidently recommended.

Another possible barrier to wider use of mHealth apps is that of digital exclusion. A significant proportion of the population lacks Internet access or has low digital literacy. These tend to be the elderly, disabled, and ethnic minorities [43]. These populations require health care the most, hence exemplifying the inverse care law [44]. This barrier is continuously being tackled through the work of the Tinder Foundation, providing online resource training to 220,000 people [42].

Furthermore, even with Internet access and a sufficient level of digital literacy so as to be open to using a healthy living app, as is the case of 37% of UK individuals, only 3% use them [45]. This highlights that more research needs to be undertaken in exploring the factors that influence individuals’ attitudes and behavioral intentions to use such apps. For example, previous research has shown that gamification, “the use of game design elements in nongame contexts” [46], can represent a highly effective way to engage users with mHealth apps [47]. Another important consideration is that, in the present study, users of not only SF but also QG stated that the lack of human contact from a trained health care worker made mobile apps less attractive as a single therapy form for them. This raises important questions in terms of the overall capabilities of CBT delivered via mobile apps. Such findings suggest the need for a study investigating both objective and subjective measures, as well as their interaction.

### Limitations and Future Research

There are a number of limitations to this study. First, because of time constraints, participants were only able to use and evaluate the apps for the duration of 1 week. Although it allowed for participants to form opinions on the themes explored, this short period prevented users from completing the programs offered by each of the 2 apps (8 weeks for QG and 4 weeks for SF), which would possibly have provided them with a more comprehensive impression of the apps and their effects. This period may also have been insufficient to determine the sustained effects of the apps. Therefore, the descriptive statistics we report are not necessarily an appropriate representation of the expected behavioral and perceptual effects which would be anticipated with the completion of the programs. Second, convenience sampling was used, where recruitment took place on a university campus, resulting in a sample of mostly university students with a mean age of 24.66 years. This may have led to a misrepresentation of the overall population, thereby bringing into question the transferability and generalizability of the conclusions. Third, the study lacked a control condition, against which the effects of each app on users’ positive behavior change could be compared, so as to evaluate these effects more accurately.

Therefore, future studies should consider using a randomized controlled trial design in a larger, more representative sample with more varied demographic characteristics, running over a longer period of time, allowing for the completion of the programs offered by each of the apps. This would produce more generalizable, conclusive, and reliable results. After this, more in-depth research could address any differences in behavioral changes elicited by the use of the app(s), as well as the effect of increased options for tailoring and personalization on measures of behavioral change and adherence. Numerous health care offerings are being digitally transformed. Yet, important questions remain about effective user engagement across different digital platforms [48,49], and the potential of digital health solutions [50] for improving people’s lives and enhancing their willingness to recommend new digital solutions to family, friends, and colleagues [51,52]. Additional research into the understanding of psychological barriers to adoption of new mHealth solutions and technologies that can inform the design and communication of new mobile health care solutions to facilitate behavior change is richly deserving.

### Conclusions

In conclusion, investigating the results of the thematic analysis carried out in this study revealed a generally more positive attitude of QG users with regard to the app’s features, design, as well as information engagement and quality, compared with SF users. QG users also reported changing their perceptions and way of thinking with respect to smoking, and noted, on average, increased willingness to use a smoking cessation app in general to manage health, as well as increased willingness to continue using their allocated app, and increased motivation to quit smoking after 1 week of app use. On average, these participants also showed changes in their smoking behavior although, of note, this was in the context of our limited sample, not allowing for the finding to be generalizable as of yet. It is possible that the root of these effects may lie in CBT, which gives users the opportunity to explore and change their thoughts and perceptions related to smoking.

This suggests that CBT has the potential to work effectively in the context of a gamified mobile app for smoking cessation; however, future research involving wider distributed samples and longer periods is required to draw more generalizable conclusions. The findings also suggest that a mobile app must be well developed, preferably with an underlying behavioral change mechanism, to promote positive perceptual and health behavior change in the context of smoking cessation. The potential of digital CBT delivered through a gamified mobile platform should be seen as a powerful tool to overcome current health care challenges.

## Abbreviations

ACT: acceptance and commitment therapy
mHealth: mobile health
NCD: noncommunicable disease
CBT: cognitive behavioral therapy
NHS: National Health Service
QG: Quit Genius
SF: NHS Smokefree
